# The Treatment of Refractory Pituitary Adenomas

**DOI:** 10.3389/fendo.2019.00334

**Published:** 2019-05-29

**Authors:** Congxin Dai, Xiaohai Liu, Wenbin Ma, Renzhi Wang

**Affiliations:** Department of Neurosurgery, Peking Union Medical College Hospital, Chinese Academy of Medical Sciences & Peking Union Medical College Beijing, Beijing, China

**Keywords:** refractory pituitary adenomas, surgical treatment, medical therapy, radiotherapy, temozolomide, targeted therapy, immunotherapy

## Abstract

Refractory pituitary adenomas (PAs) are defined as aggressive-invasive PAs characterized by a high Ki-67 index, rapid growth, frequent recurrence, and resistance to conventional treatments. It is notoriously difficult to manage refractory PAs because the efficacy of current therapeutic options is limited. The purpose of this review is to address currently employed and promising therapeutic strategies for the treatment of refractory PAs. Except for prolactinomas, neurosurgery is the first-line option, but most refractory PAs often recur or re-grow after initial surgery and require further treatments. Medical therapy, radiotherapy and re-operation are explored when surgery has failed to completely resect tumors; however, refractory PAs are usually resistant to these treatments. As a salvage treatment, temozolomide (TMZ) has shown promising results and is currently used for all types of refractory PAs. However, not all refractory PAs are responsive to TMZ treatment, and some of these PAs are resistant to TMZ. Although targeted therapies such as vascular endothelial growth factor, epidermal growth factor and mTOR inhibitors have also been used to treat refractory PAs, the effectiveness of these targeted therapies is still not known due to a lack of data from randomized prospective trials. As a novel therapeutic method, cancer immunotherapy is a promising strategy for the treatment of refractory PAs, but further preclinical research and clinical trials are needed to assess the efficacy of this new approach. In summary, early identification and a multidisciplinary approach are required to treat refractory PAs.

## Introduction

Most pituitary adenomas (PAs) are benign and exhibit slow expansive growth; however, approximately 35% of pituitary adenomas are invasive and some of them exhibit more aggressive clinical behavior with high rates of recurrences (1, 2). In the clinic, a subset of aggressive-invasive PAs characterized by a high Ki-67 index, rapid growth, frequent recurrence, and resistance to conventional treatments is defined as refractory PAs (3). The definition of refractory pituitary adenomas includes radiological finding, histopathological, and clinical features. Trouillas et al. (4) also performed a retrospective multicentric study and proved that Ki-67 and other proliferation parameters could be used to define aggressive tumors, which is similar to our proposal. They classified PAs 5 grades (grade 1a: non-invasive, 1b: non-invasive and proliferative, 2a: invasive, 2b: invasive and proliferative, and 3: metastatic) based on tumor size, type, invasion, P53 and markers of the cell cycle (Ki-67). After 8-year follow-up, they found that invasive and proliferative tumors (grade 2b) had a poor prognosis compared to non-invasive tumors. These refractory PAs often have a very poor prognosis and even have a fatal outcome. However, there is no general agreement on how to manage patients with refractory PAs. For neurosurgeons and clinicians, it is difficult to choose the optimal therapeutic options in the treatment of refractory PAs. Therefore, to improve the prognoses of these patients, it is very important and necessary to review the emerging treatments for refractory PAs.

## Management of Refractory PAs

### Surgical Treatment

Typically, multimodal approaches are required to manage refractory PAs. Other than prolactin-secreting adenomas (PRL-omas), which should first be treated with dopamine agonists (DAs), the primary treatment option is usually surgery. Most refractory PAs are largely invasive and infiltrate the adjacent tissues, surgery is usually unable to cure or control them (5). However, the therapeutic goals of surgery are maximum reduction in tumor mass, decompression of visual pathways, best possible reduction in hormonal oversecretion, amelioration of clinical symptoms and release of complications (6). After the primary operation, refractory PAs always recur or re-grow early, and need further surgical intervention. Although repeated surgery seldom achieves complete excision. it is still necessary to relieve compressive symptoms.

Dopamine agonists are the first treatment of choice for prolactinomas, however, resistance to dopamine agonists occurs in a subset of patients with PRL-omas.

TSS surgery is recommended for an important therapeutic option for these resistant prolactinomas. In a retrospective study, Primeau et al. (7) analyzed outcomes of patients with a prolactinoma treated by TSS, and found that postoperative remission was obtained in 63% of microprolactinomas, 60% of noninvasive macroprolactinomas, and none of the invasive macroprolactinomas. However, after surgical remission, a recurrence of hyperprolactinemia was observed in one-third of prolactinoma patients after a median follow-up period of 36 (7–164) months. Another retrospective study indicated that normalization of prolactin occurred in 87% of patients with microadenomas and in 56% of macroadenomas after surgery, and recurrence of hyperprolactinemia occurred in 13% of them at 10 years follow-up (8).

Although repeat TSS is less effective for residual and recurrent pituitary adenomas, it may still be beneficial for many patients by reducing mass effect or adenoma burden to improve the effectiveness of adjuvant therapies. Vargas (9) carried out a retrospective study on treatment outcome of 485 Patients with NFPA, the results indicated that after first surgery, 27.2% (127/466) found to have tumor persistence, and second pituitary surgery was performed in these 127 patients, 23.6% (30/127) of them were documented with tumor persistence and need third operation. In 2016, a systematic review and evidence-based guideline for residual or recurrent NFPAs was published by the Congress of Neurological Surgeons, and repeat resection is listed as a level III recommendation for the treatment of symptomatic recurrent or residual NFPAs (10). Mercado (11) reported that the presence of extension into the cavernous sinus and of an adenoma remnant after initial surgery associated with recurrence. Espinosa De Los Monteros et al. (12) also evaluated the outcome of surgical reintervention in patients with active acromegaly, and found that only 9% (5/53) of patients achieved complete biochemical cure. Almeida et al. (13) assessed the outcomes of reoperation for patients with residual or recurrent growth hormone-secreting pituitary adenoma at the authors' institution, and no statistically significant difference was found in disease control rates between the patients who underwent reoperation and first-time neurosurgery. They further systematically reviewed 161 reoperations and 2,189 first-time surgery cases retrieved from 29 papers and found that reoperation and first-time surgery had similar control rates for microadenomas but that reoperation was related to substantially lower control rates for macroadenomas (27.5%) and tumors invading the cavernous sinus (14.7%). Mendoza et al. (14) evaluated the long-term efficacy of the different secondary interventions for persistent and recurrent Cushing's disease, and found that early remissions were observed in 66.6% of the re-operated patients, and a long-lasting remission was achieved in only 33.3% patients. However, the rate of complications including transient diabetes insipidus, and arachnoid tear related to surgical reintervention was raised with the increasing times of operation. Because of the relatively small numbers of reported recurrences of CD and incompletely reported complications, whether significant differences exist in complications between primary and repeat resections is still controversial (15).

The comparison of microscopic and endoscopic approaches for recurrent or residual PAs remains controversial. Heringer et al. (16) performed a meta-analysis to evaluate the effect of repeated TSS in recurrent or residual PAs and found that half of secreting tumors and more than half of nonfunctioning PAs (NFPAs) could achieve remission after surgery, and that there is no difference between the endoscopic and microscopic approach. However, Esquenazi et al. performed another meta-analysis to compare the effects of endoscopic and microscopic TSS on recurrent and/or residual PAs, and found that endoscopic surgery led to modest increases in resection rates on residual or recurrent adenomas (17). Do et al. (18) retrospectively analyzed 61 patients with recurrent or residual PAs who underwent endoscopic endonasal surgery and found that gross total resection was achieved in 31 patients (51.7%), indicating that the endoscopic endonasal approach is a safe and effective option for recurrent PAs. The results from another meta-analysis performed by Li and colleagues also indicated that endoscopic surgery is related to higher gross tumor removal and lower incidence of complications in patients with PAs (19). Based on previous studies and our experience, endoscopic surgery is better than microscopic surgery for recurrent PAs; however, these findings need to be verified by large-scale prospective randomized controlled trials.

Therefore, maximum tumor resection, while preserving nerve function, is necessary to achieve local control and decompress vital structures for refractory PAs with compressive symptoms.

### Radiation Therapy

Despite the success of TSS or maximum tumor resection, most refractory PAs will re-grow or recur; therefore, other therapeutic approaches are usually needed. If surgical and/or medical therapy failed to control the tumor growth, radiation therapy (RT) is currently the next treatment option. There are several RT options for patients with refractory PAs. Conventional external beam radiotherapy (EBRT) has been used to treat pituitary adenomas for several decades and has shown good clinical safety and efficacy. However, EBRT can result in significant complications, such as hypopituitarism, cognitive function deficiency and cerebrovascular disease (20). In recent years, EBRT has largely been replaced by the stereotactic radiosurgery (SRS) and fractionated stereotactic radiotherapy (FSRT). Stereotactic radiosurgery (SRS) is the delivery of a single high dose of radiation under conditions of accurate positioning. As new methods of radiation delivery, SRS and FSRT could minimize these complications. Recently, SRS has been gaining popularity because it minimizes the exposure of normal brain tissue to radiation. SRS has been preferred over EBRT because of the convenience of single-day therapy and the potential for a faster effect on the tumor (21). A variety of SRSs, including Gamma Knife radiosurgery (GKRS), CyberKnife (CK) and proton beam RT, are available to deliver stereotactic RT.

Comparing EBRT and SRS may help guide decision making for patients with residual or recurrent pituitary tumors. Kong et al. (22) compared the efficacy and safety of SRS and EBRT for the treatment of 125 patients with PAs. Although no significant difference was found in either biochemical remission or tumor growth control, the time to biochemical remission after SRS was much shorter than that following EBRT (26 vs. 63 months).

To better understand the effects of SRS for Cushing's disease (CD), 23. Mehta et al. (23) performed an international, multicenter, retrospective cohort analysis that included 278 patients with CD who received SRS and found that the overall rate of durable control of hypercortisolism was 64% at 10 years; in addition, adverse radiation effects including hypopituitarism (25%) and cranial neuropathy (3%) were observed. However, recurrences occurred in 18% of the patients with initial cortisol normalization. In one prospective study, GKRS-induced hypopituitarism occurred in 58.3% of patients with a recurrent or residual acromegaly or CD (24). CK is a frameless image-guided stereotactic device that is becoming increasingly incorporated into the treatment of refractory pituitary tumors. A retrospective study indicated that 57.1% of patients with persistent or recurrent CD achieved biochemical remission after treatment with CK, and few complications were observed (25). FSRT is also frequently employed for the treatment of residual or recurrent PAs. Minniti et al. analyzed the effects of FSRT in sixty-eight patients with large residual or recurrent NFPAs and found that the 5- and 10-year actuarial local control rates were 97 and 91%, respectively. However, the incidence of new hypopituitarism was 40% at 5 years and 72% at 10 years (26). Another study by Sheehan and colleagues indicated that GKRS treatment of patients with CD after failed TSS could result in 63% of the patients achieving normal 24-h urinary free cortisol (UFC) levels; however, 16% of the patients obtained new endocrine deficiencies (27). Losa et al. (28) conducted another retrospective analysis of patients with pituitary adenoma treated by GKRS and found that patients with NFPAs have more frequent recurrence of disease than patients with hormone-secreting adenomas (9.6 vs. 4.8%). The 10-year progression-free survival in patients with NFPAs was 78.7%, which is much lower than that in patients in hormone-secreting adenomas (93.3%). The data on re-irradiation for recurrent PAs are limited. To evaluate the safety and efficacy of re-irradiation for recurrent PAs, Verma et al. (29) retrospectively analyzed the outcomes of 15 patients receiving re-irradiation including fractionated RT and SRS. The results indicated that actuarial local control rates at 2 and 5 years were 80 and 58%, respectively. However, the 5-year rate of radiation-induced optic neuropathy was 9%, the 5-year rate of temporal lobe necrosis was 28%, and four patients (27%) ultimately developed pituitary carcinoma after re-irradiation.

To compare the efficacy and safety of SRS and FSRT for treatment of PAs, X. Li performed a meta-analysis and founded that no significant differences were found in efficacy measures, such as disease control rate and endocrine cure rate, or complications, such as hypopituitarism and visual disturbance rate between SRS and FSRT. Therefore, both SRS and FSRT are comparable efficacious and safe for patients with Pas (30).

According to the Clinical Practice Guidelines initiated by European Society of Endocrinology (31), SRS is suited for the tumor <3 cm in diameter and the tumor should be at least 3–5 mm distant from the optic chiasm. Otherwise, fractionated EBRT may be the only option. Furthermore, to avoid high dose radiation of healthy tissue, EBRT also should be preferred for tumors with irregular anatomy, including suprasellar or brainstem extension and diffuse local infiltration. Refractory PAs are not candidates for stereotactic RT because of tumor size (>3 cm) or tumor location near the optic apparatus and brainstem (<5 mm) (32). Risks associated with RT including hypopituitarism, optic neuropathy and other cranial neuropathies should be considered and avoided.

To conclude, both conventional EBRT and SRS have shown a good tumoristatic effect on residual or recurrent pituitary adenomas. In any of its modalities, RT constitutes a relatively cheap option for the treatment of these residual tumors.

### Medical Therapy

Medical therapy plays an increasingly important role in the treatment of PAs. In secreting pituitary tumors, medications not only inhibit pituitary tumor growth but also control biochemical oversecretion in some cases. In recent years, there is accumulating evidence indicated that many medications used for functional tumors may be beneficial for residual or recurrent NFPAs.

### PRL-omas

For patients with PRL-omas, DAs such as bromocriptine or cabergoline are the primary treatment and achieve successful treatment of these adenomas (33).

Cabergoline was more effective than bromocriptine in normalizing serum prolactin levels, shrinking prolactinomas, and controlling symptoms associated with hormone excess (34). E. Espinosa reported that Cabergoline treatment could result in the normalization of PRL levels in 68% and in the reduction of 50% in tumor volume in 87% of the giant PRL-oma patients (35).

In most of PRL-omas, treatment with DAs, normalization of prolactin and tumor shrinkage could be achieved and do not require other surgical or radiotherapy interventions. However, a subset of patients with PRL-omas will resistant to dopamine agonists. Although there is no clear consensus on the definition of Resistant prolactinomas. Resistant prolactinomas is generally defined as a failure to normalize prolactin levels or inability to induce tumor shrinkage despite the administration of more than 15 mg of bromocriptine daily for at least 3 months, or more than 1.5–2.0 mg of cabergoline weekly (36). There are several possible mechanisms of dopamine agonist resistance in prolactinomas. Most focus on decreased dopamine receptor expression, and alterations in cellular signaling factors downstream of dopamine receptors. The previous study had shown that resistance to dopaminergic agonists seems to involve defects in D2 dopamine receptor expression (37). Caccavelli et al. (38) furthermore found that decrease in D2 dopamine receptors is associated with a decrease in G alpha i2 expression, and indicated that the resistance to dopaminergic agonists is not just due to decreased cell surface D2 receptors, but is also caused by decreased expression of the downstream of the D2 receptor (G alpha i2 inhibitory G protein). Shimazu et al. (39) also had proved that the reduction in D2L isoform mRNA levels is correlated with resistance of prolactinoma to dopamine agonists. PRDM2 is a retinoblastoma interacting zinc-finger protein, Gao et al. (40) had shown that PRDM2 downregulation may play a role in dopamine-agonist resistance.

There are a few therapeutic alternatives for DA-resistant prolactinomas, E. Sosa-Eroza reported that addition of octreotide to ongoing cabergoline treatment resulted in significant reductions in PRL levels and tumor volume in 2 out of 5 patients (41). Moreover, DAs treatment does also have some side effects, such as nausea, vomiting, headache, and dizziness or vertigo. Rapid tumor shrinkage caused by DAs could result in leakage of cerebrospinal fluid (CSF), which is a risk of DAs as well.

### Growth Hormone-Secreting PAs (GH-omas)

TSS is recommended as the primary option for GH-omas; however, biochemical control could be achieved for only 40–60% of invasive GH-omas, even with TSS performed by expert pituitary neurosurgeons (42). For those patients for whom surgical approaches have failed to control the disease, somatostatin analogs (SSAs), such as octreotide and lanreotide. are the next step option. The long-acting formulation of pasireotide has greater efficacy than octreotide and lanreotide and could be tried if octreotide and lanreotide are not effective (43). The SSAs could achieve biochemical control in only 20–35% of patients with GH-omas (44). Resistance to SSAs may be defined as a failure to achieve biochemical control criteria (GH< 1.0 μg/L and a normal age-adjusted IGF-1) and increase in tumor size or tumor shrinkage <20% compared with baseline volume after at least 12 months of treatment with SSAs (45).

Several markers have been shown to predict responsiveness to SRLs in acromegaly. These main predictors include somatostatin receptor (SSTR) expression, densely or scarcely granulated tumors, AIP and Ki-67 (46). It has been shown that resistance to SRLs is related to reduction of somatostatin receptor (SSTR) density or to a differentiated expression of SSTR subtypes (47). It is widely suggested that the response to SRLs treatment in acromegaly correlates with expression of the SSTR2 and SSTR5 subtypes (48–50). Besides SSTRs expression, AIP (aryl hydrocarbon receptor-interacting protein) also have been demonstrated to be associated with response to SRLs treatment. The previous studies indicated that low AIP expression in sporadic correlate to a poor response to SRLs (51, 52). Moreover, densely granulated GH-omas are highly responsive to SRLs than the sparsely granulated adenomas (53). Furthermore, Ki-67 also has been reported as a predictor of response to SRLs in GH-omas, which is independent of SSTR2 expression and relates to cytokeratin patterns (54). Therefore, personalized therapy based on these predictors could increase treatment efficacy with more rapid disease control and cost reduction.

Cabergoline also exhibited a therapeutic effect on GH-omas and could achieve biochemical control in approximately one-third of these patients; combining cabergoline with SSAs can further improve therapeutic success in 52% of patients with GH-omas (55). The most common adverse effects of SSAs are worsening of glucose tolerance and diabetes, gallbladder stones and sludge, abdominal cramps, flatulence, and diarrhea. Pegvisomant is a GH receptor antagonist that can decrease IGF-1 levels in patients with GH-oma. Although pegvisomant could normalize IGF-1 levels in 60–80% of patients, it cannot decrease GH levels or shrink tumor size because it does not have any direct effect on the tumors (56, 57). Surgical debulking of pituitary tumors, radiotherapy and radiosurgery are reserved for patients who are resistant or intolerant to medical treatment.

### Adrenocorticotropin Hormone (ACTH)-Secreting PAs

As the initial preferred treatment, TSS could achieve a high (70–85%) rate of remission in ACTH-secreting PAs; however, tumors may recur in up to 25% of patients and require further therapy (58). If surgery fails, then options including medical therapy, pituitary irradiation, or bilateral adrenalectomy are used. Several medications have been reported to normalize cortisol levels and improve morbidity and mortality. Currently available medical treatments for patients with ACTH-secreting PAs include steroidogenesis inhibitors, centrally acting agents, and a glucocorticoid receptor antagonist.

Pasireotide and cabergoline can directly act on ACTH-secreting PAs via inhibiting ACTH production. As a novel multireceptor ligand SSA with a high binding affinity for somatostatin (SST) receptors, pasireotide could act directly on ACTH-secreting PAs to inhibit ACTH production. Pasireotide can normalize cortisol levels in 19% of patients with CD. However, pasireotide also caused a worsening of glucose tolerance in 73% of these patients (59). Cabergoline is a newer dopamine agonist and has high affinity for the dopamine receptor subtype 2, which is expressed in most ACTH-secreting PAs. Recent studies showed that cabergoline can normalize cortisol levels in approximately one-third of patients with CD (60).

Steroidogenesis inhibitors currently in use include ketoconazole, metyrapone, mitotane, and etomidate (61). Although ketoconazole could normalize cortisol levels in approximately 50% of patients with ACTH-secreting PAs, 20.5% of them could not continue the treatment due to poor tolerance including liver toxicity and gastrointestinal complaints (62).

Metyrapone, a steroidogenesis inhibitor, could convert 11-deoxycortisol to cortisol and control cortisol levels in 50–76% of patients with CD. However, metyrapone use also results in adverse events including mild gastrointestinal upset and dizziness in 25% of patients (63). Mitotane is an adrenolytic agent used in the treatment of adrenocortical carcinoma, and it has recently been approved for the treatment of CD. Long-term treatment with mitotane can cause remission in 72% of patients with CD, but gastrointestinal and neurologic side effects are very common (64). If all the above measures fail, the intravenous imidazole derivative etomidate could be useful. Etomidate can block several steps in cortisol synthesis and may be used as first-line treatment for severe hypercortisolism in patients with severe CD (65). Recent guidelines suggest that etomidate may be useful for patients with life-threatening hypercortisolemia who cannot take oral medications (66).

Mifepristone is a glucocorticoid receptor antagonist and antiprogestin. Mifepristone does not inhibit cortisol synthesis but directly antagonizes its effects via blocking the cortisol glucocorticoid receptor and the progesterone receptor. Mifepristone is useful for the control of diabetes or glucose intolerance caused by hypercortisolism. Mifepristone could improve diabetes and hypertension in 60 and 40% of patients with Cushing's syndrome, respectively, and 87% of these patients showed significant clinical and quality-of-life improvements (67). However, ACTH levels increased dramatically in 72% of patients, and cortisol levels remained unchanged or increased during treatment. The common side effects of mifepristone were cortisol insufficiency symptoms including fatigue, nausea, headache, low potassium, arthralgia, vomiting and edema (67). Combinations of some of these medications may be more effective than single agents in some patients; however, the adverse events should be considered as well.

Osilodrostat, a potent oral 11b-hydroxylase inhibitor, has been proved that could normalize urinary free cortisol (UFC) in patients with CD. The result from a Phase II clinical trial indicated that Osilodrostat treatment reduced UFC in all CD patients; 78.9 % (n/N = 15/19) had normal UFC at week 22 (68). Roscuvitine is a CDK2 inhibitor, Liu et al. have proved that roscuvitine could inhibit human pituitary corticotroph tumor ACTH by targeting the cyclinE/E2F1 pathway (69). Liu et al. (69) demonstrated that USP8·STAM complex as a protective mechanism regulating early endosomal sorting of EGFR between pathways destined for lysosomal degradation and recycling.

The previous studies have shown retinoic acid could inhibits ACTH secretion *in vitro* by inhibiting the transcriptional activity (70). Pecori Giraldi et al. (71) evaluated the efficacy and safety profile of retinoic acid in patients with CD, and found that retinoic acid proved beneficial and well tolerated in 71% of (5/7) patients with CD. Vilar et al. (72) also proved that combination of isotretinoin (13-cis-retinoic acid) with cabergoline may occasionally be more effective than either drug alone.

Although medical treatment in ACTH-secreting PAs has achieved rapid advancement, some refractory CD cases are still resistant to current medications, and bilateral adrenalectomy would be required (72). Adrenalectomy could achieve immediate cessation of hypercortisolism but will result in life-long adrenal insufficiency and Nelson syndrome (73).

### NFPAs

Because of the lack of clinical symptoms caused by secreted hormones, most NFPAs are large at diagnosis due to symptoms related to mass effect. Complete resection is not always possible, especially in large invasive macroadenomas. Adjuvant radiation can effectively prevent residual tumor growth, but re-growth or recurrence often occur; therefore, effective medical treatment for NFPA is necessary.

DAs are the first-line choice of treatment of PRL-omas via the activity of dopamine receptors. Most NFPAs also express dopamine receptor 2 (D2R), which may be a potential therapeutic target for NFPAs (74). Previous studies have suggested that DAs are effective in the treatment of NFPAs. Greenman et al. (75) evaluated the effect of DAs on postsurgical NFPAs; their results showed that after preventive treatment with DAs, 38% of patients achieved a decrease, 49% showed no change, and 13% showed an increase in tumor size,. Further, DAs induced tumor shrinkage or restrained tumor growth in over 58% of patients who already have tumor enlargement.

SST receptor ligands are widely used for medical treatment of acromegaly and thyroid-stimulating hormone (TSH)-secreting tumors. SST inhibits tumor cell growth arrest and hormone secretion through binding of five different SST receptors.

NFPAs express different SST receptors as well, suggesting that SST receptor ligands may be an effective medical treatment for NFPAs (76). Colao et al. (77) summarized case reports and small uncontrolled studies and found that in patients treated with octreotide, tumor volume was decreased in 12%, increased in only 5% and remained unchanged in 83% of these patients. In a case-control study conducted by Fusco et al., 26 preselected patients with positive tumor uptake in SST receptor scintigraphy were treated with the long-acting SST LAR; tumor size increased in 19% of the patients, and the remaining patients remained stable (78).

## Future Perspectives and Conclusions

Temozolomide (TMZ) is an orally administered alkylating chemotherapy that readily crosses the blood-brain barrier. TMZ is considered the standard treatment in the management of gliomas. In 2006, the first successful treatment of PA with TMZ was reported (79, 80), and TMZ treatment has also been widely used for patients with refractory PAs and carcinomas (81). In 2017, the European Society of Endocrinology Clinical Practice Guidelines recommended the use of TMZ monotherapy as first-line chemotherapy for aggressive PAs and pituitary carcinomas (31). To our knowledge and to date, approximately 160 cases of pituitary tumors treated with TMZ have been reported. The data form a Multicenter retrospective study indicated a 51.2% response rate to TMZ, with an improved survival among responders despite frequent relapses (82). In a recent meta-analysis, the 5-year OS and 5-year progression free survival for aggressive pituitary adenomas treated with TMZ was 57.4 and 21.9% respectively (83).

However, most of refractory PAs failed to respond to TMZ and even acquired TMZ resistance after an effective response to TMZ (82). Therefore, it is important to enhance the efficacy of TMZ and overcome the resistance to TMZ. The presence of certain molecules in pituitary tumors, such as MGMT and MSH6, has been associated with temozolomide response (84).

Epidermal growth factor is a cell growth factor that regulates cell proliferation and hormone production in pituitary tumors (85). EGFR is overexpressed in prolactinoma and ACTH-secreting PAs, which may offer a potential therapeutic target for refractory pituitary tumors (86, 87). As an EGFR inhibitor, gefitinib has shown antiproliferative and apoptotic effects in corticotroph tumor cells *in vitro* (86). Lapatinib, a dual HER2/EGFR inhibitor, was shown to suppress both the expression and secretion of PRL mRNA to a greater extent than gefitinib in an animal model of prolactinoma (88). Although further clinical trials are needed, preclinical data suggest that the EGFR pathway may be an effective therapeutic target for patients with refractory pituitary tumors.

Vascular endothelial growth factor (VEGF) is a potent angiogenic factor in pituitary tumors. Previous studies have indicated that angiogenesis is associated with adenoma development, local invasion, and recurrence (89–91). Several studies reported that angiogenesis decreases tumor sizes in humans and experimental pituitary tumors (89, 92, 93)^.^ Ortiz et al. reported the first case of a bevacizumab-treated pituitary carcinoma with long-term stabilization of disease in 2012 (94). Touma et al. also presented one case of pituitary carcinoma treated successfully with concurrent chemoradiation therapy and bevacizumab with long-term follow-up (95). However, the role of anti-VEGF therapy in pituitary tumors is still controversial due to a lack of large-scale clinical trials.

PI3K/AKT/mTOR cascades are key signaling pathways in the tumorigenesis of PA (96). Previous studies reported that the PI3K/AKT/mTOR pathway is upregulated and overactivated in PAs, indicating an important role in tumor formation and progression of PAs (97–99). Inhibition of the PI3K/mTOR signaling pathway not only displays antitumor efficacy against pituitary tumors (100, 101) but also sensitizes PA cells to radiotherapy and chemotherapy (102, 103). Donovan et al. reported one patient with pituitary carcinoma, which is refractory to multiple surgery, radiation and chemotherapy, who achieved clinical improvement and stability for more than 6 months after treatment with an mTOR inhibitor and radiation (104).

As a promising therapeutic approach, cancer immunotherapy has been attracting increasing attention recently. To date, immunotherapy has been used for the treatment of many tumors, including glioma, lung cancer, melanoma, prostate cancer, and B cell lymphoma (105). In 2007, Hazrati et al. reported one case of a prolactinoma treated successfully with immunotherapy for the first time (106). Lu et al. have reported that CD68+ macrophage infiltration is associated with PA size and invasiveness, indicating that immunotherapy may be useful to restrict the tumor enlargement and invasiveness (107). Blocking the interaction between the programmed cell death (PD-1) protein and one of its ligands, programmed death ligand 1 (PD-L1), is a novel strategy for cancer immunotherapy. The expression of PD-L1 is positively correlated with improved responses to anti-PD-1/PD-L1 blockade in many cancers (108). Mei et al. reported that the expression of PD-L1 is significantly higher in human functioning adenomas compared to that in nonfunctioning adenomas, suggesting the existence of an immune response to pituitary tumors (109). Therefore, these studies raise the possibility of considering immunotherapy for refractory PAs.

## Conclusion

In summary, refractory pituitary tumors are usually unresponsive to different therapies and have a poor prognosis. To improve the survival of patients, early identification and a multidisciplinary approach is required. Although various treatment options are available to manage these refractory pituitary tumors, the efficacy is limited. Therefore, new therapeutic approaches and randomized clinical trials are needed. It is hoped that further research may clarify the tumorigenesis and pathogenesis of refractory pituitary tumors and that additional alternative treatments may be developed for these tumors.

## Author Contributions

All authors listed have made a substantial, direct and intellectual contribution to the work, and approved it for publication.

### Conflict of Interest Statement

The authors declare that the research was conducted in the absence of any commercial or financial relationships that could be construed as a potential conflict of interest.
